# The Genetic Risk for COVID-19 Severity Is Associated With Defective Immune Responses

**DOI:** 10.3389/fimmu.2022.859387

**Published:** 2022-05-12

**Authors:** Yunus Kuijpers, Xiaojing Chu, Martin Jaeger, Simone J. C. F. M. Moorlag, Valerie A. C. M. Koeken, Bowen Zhang, Aline de Nooijer, Inge Grondman, Manoj Kumar Gupta, Nico Janssen, Vera P. Mourits, L. Charlotte J. de Bree, Quirijn de Mast, Frank L. van de Veerdonk, Leo A. B. Joosten, Yang Li, Mihai G. Netea, Cheng-Jian Xu

**Affiliations:** ^1^ Centre for Individualised Infection Medicine, CiiM, a joint venture between the Hannover Medical School and the Helmholtz Centre for Infection Research, Hannover, Germany; ^2^ TWINCORE, Centre for Experimental and Clinical Infection Research, a joint venture between the Hannover Medical School and the Helmholtz Centre for Infection Research, Hannover, Germany; ^3^ Department of Genetics, University Medical Center Groningen, University of Groningen, Groningen, Netherlands; ^4^ Department of Internal Medicine and Radboud Institute for Center for Infectious Diseases, Radboud University Medical Center, Nijmegen, Netherlands; ^5^ Núcleo de Pesquisa da Faculdade da Polícia Militar (FPM) do Estado de Goiás, Goiânia, Brazil; ^6^ Department for Genomics and Immunoregulation, Life and Medical Sciences Institute (LIMES), University of Bonn, Bonn, Germany; ^7^ Department of Gastroenterology, Hepatology and Endocrinology, Hannover Medical School, Hannover, Germany

**Keywords:** genome-wide association studies, COVID-19, genotyping, cytokine, single-cell

## Abstract

Recent genome-wide association studies (GWASs) of COVID-19 patients of European ancestry have identified genetic loci significantly associated with disease severity. Here, we employed the detailed clinical, immunological and multi-omics dataset of the Human Functional Genomics Project (HFGP) to explore the physiological significance of the host genetic variants that influence susceptibility to severe COVID-19. A genomics investigation intersected with functional characterization of individuals with high genetic risk for severe COVID-19 susceptibility identified several major patterns: i. a large impact of genetically determined innate immune responses in COVID-19, with ii. increased susceptibility for severe disease in individuals with defective cytokine production; iii. genetic susceptibility related to ABO blood groups is probably mediated through the von Willebrand factor (VWF) and endothelial dysfunction. We further validated these identified associations at transcript and protein levels by using independent disease cohorts. These insights allow a physiological understanding of genetic susceptibility to severe COVID-19, and indicate pathways that could be targeted for prevention and therapy.

## Introduction

The novel coronavirus disease 2019 (COVID-19), caused by the severe acute respiratory syndrome coronavirus 2 (SARS-CoV-2) (1, 2), firstly emerged in late December 2019. Since then, it spread worldwide causing a major pandemic with severe consequences for the health of millions of individuals and major economic disruption. Even though much has been learned about the pathophysiology of the disease, and immune-based treatments, including dexamethasone (3) and anti-IL-6/IL-1 therapeutic strategies (4–6), proven to be effective, the prognosis is still poor in many patients with severe disease. Therefore, there is an urgent need to better understand the exact host-pathogen interactions leading to increased severity and mortality to design additional prophylactic and therapeutic strategies in the future (7, 8), including in a personalized manner.

The severity of SARS-CoV-2 infection is highly variable and ranges from asymptomatic to mild disease and to severe Acute Respiratory Distress Syndrome with a fatal outcome (9). However, the causes for this broad variability in disease outcome between individuals are largely unknown (10, 11). A recent study indicates that human host factors rather than viral genetic variation affect COVID-19 severity outcome (12). Additionally, clinical and epidemiological data have shown that old age, male sex, and chronic comorbidity are associated with higher mortality (13, 14). The first genome-wide association study in individuals with European genetic ancestry has identified several chemokine receptor genes, including *CCR9*, *CXCR6*, and *XCR1* and the locus controlling the ABO blood type to be associated with severe symptoms of COVID-19 (15). Nevertheless, little is known about the mechanisms through which these genetic variants influence COVID-19 severity. For example, several competing hypotheses can be envisaged for the involvement of immune genes in susceptibility to severe COVID-19. On the one hand, it may be hypothesized that genetic risk for severe COVID-19 is associated with defective innate immune responses that would allow viral multiplication with high viral loads; on the other hand, the opposite hypothesis may also be true, with an exaggerated genetically-mediated cytokine production might be responsible for the late phase hyperinflammation and poor outcome. A genetic study alone cannot respond to this crucial question, which would have significant consequences for the approach to prophylaxis and therapy.

**Figure 1:**
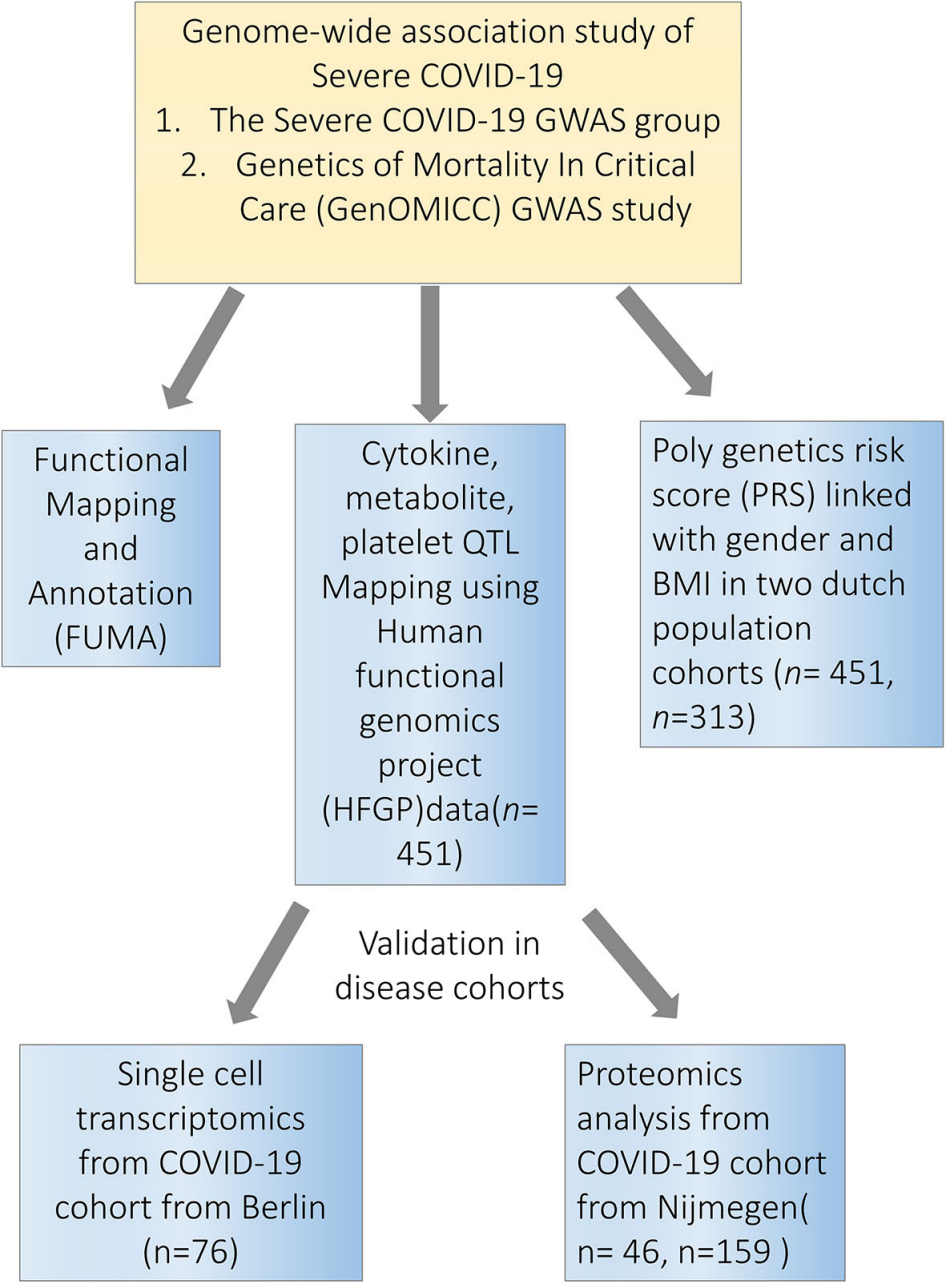
Study overview. Firstly, we performed a functional mapping and annotation (FUMA) to link COVID-19 SNPs to gene expression and identified important pathways and tissues contributing to the pathophysiology of COVID-19. Secondly, we utilized the cytokine quantitative trait loci (QTL), metabolite QTL and plate QTL from Human Functional Genomics Projects (HFGP) 500FG data (n=451) to test if specific loci are associated with immune functions. Thirdly, we linked PRS score with gender and BMI in 500FG (n=451) and 300BCG (n=313) cohorts. Lastly, we validated our findings in disease cohorts in single-cell transcriptomics data from Berlin (n=76), and proteomics data from Nijmegen (n=46, n=159). SNPs, single-nucleotide polymorphisms.

Considering the arguments above, in the present study, we used the resources from the Human Functional Genomics Project (HFGP) (16, 17) and assessed the impact of COVID-19 associated genetic polymorphisms on the variability of immune responses at the population level (**Figure 1**). We validated our findings using single-cell transcriptomics and proteomics data from two independent COVID-19 cohorts. This study helps us understand how genetic variability is related to disease susceptibility through the regulation of immune responses and endothelial function.

## Materials and Methods

### Study Cohort

In the present study, 451 and 313 healthy European individuals from the 500FG and 300BCG cohort (18) of the Human Functional Genomics Project (HFGP) (17)were recruited separately. European ancestry of all participants was confirmed with genotype measurement. Within 500FG cohort, immune cell counts, cytokine production upon stimulations, platelets, globulins, and gut microbiome were measured (For detailed information see (16, 17, 19, 20). The basic characteristics of 300 BCG participant in the BCG vaccination study (18, 21) are shown in **Table S14**. Within 300 BCG cohort, blood was collected before vaccination and cytokine production was measured upon *ex-vivo* stimulation of PBMCs with microbial stimuli.

### Genotype Quality Control and Imputation

Genotyping on samples from 500FG and 300BCG was performed using Illumina humanOmniExpress Exome-8 v1.0 SNP chip Calling by optiCall 0.7.0 with default settings. All individuals of non-European ancestry, ambiguous sex, call rate ≤ 0.99, excess of autosomal heterozygosity (F<mean-3SD), cryptic relatedness (π>0.185) were removed. SNPs with a low genotyping rate (<95%), with low minor allele frequency (<0.001), deviation from Hardy-Weinberg equilibrium (p<10^-4^) were excluded. The detailed QC steps have been published in reference (17). Genotype data of 500FG and 300BCG were imputed separately. The imputation was performed on the Michigan imputation server (22). The cohorts were phased using Eagle v2.4 with the European population of HRC 1.1 2016 hg 2019 reference panel (22). After imputation, variants with a MAF < 0.01, an imputation quality score R2 < 0.5, or a Hardy-Weinberg-Equilibrium P < 10^-12^ were excluded. All quality control steps were performed using Plink v1.9. After imputation and quality control, 451 individuals from 500FG and 313 from 300BCG were considered for downstream analyses.

### Immune Parameter Quantitative Trait Locus (QTL) Profiles

We acquired summary statistics of cytokine QTLs (17), cell proportion QTLs (19) and circulating mediators and metabolite QTLs (23) from our previous studies performed with 500FG. Metabolites were measured on the Brainshake Metabolomics/Nightingale Health metabolic platform. These samples were processed following the standard automated protocol provided by Nightingale’s technology (Finland), and blood metabolites were quantified in absolute concentrations (e.g., mmol/L) and percentages using nuclear magnetic resonance (NMR) spectroscopy. We performed QTL mapping for circulating mediators and platelet traits in 500FG using an R package MatrixeQTL (24) The measurement of circulating mediators including IL-18BP, resistin, leptin, adiponectin, alpha-1 antitrypsin (AAT), and IL-18 have been described previously (16). Platelet traits (25) include thrombin-antithrombin complex (TAT), beta-thromboglobulin total, beta-thromboglobulin, fibrinogen binding, collagen-related peptide (CRP) P-selectin, CRP fibrinogen, ADP P-selectin, ADP fibrinogen, P-selectin, platelet−monocyte complex, total platelet count, and von Willebrand factor (VWF). The circulating mediator levels and platelet traits were log2 transformed. A linear model was applied to the platelet data and genetic data by taking age and sex as covariates. We considered p-value < 5×10^-8^ to be genome-wide significant.

### Colocalization Analysis

We performed co-localization analysis (26) to look at the overlapping profile between molecular QTLs, COVID-19 GWAS, and other GWAS profiles using the R package ‘coloc’.

### Functional Analysis of Genomic Loci

We used the FUMA pipeline (27) to identify genes linked to COVID-19 with severe respiratory failure. FUMA identified significant independent SNPs as variants with P < 1×10^-5^ that were independent of each other using an LD threshold of r2 < 0.6. Within these significant independent SNPs, variants lead SNPs are identified as the most significant variants that are independent using an LD threshold of r2 < 0.1. We mapped genes to these SNPs based on their genomic position allowing for a maximum distance of 10kb. In addition to this, genes were also mapped based on eQTL effects. Genes were selected based on significant SNP-gene pairs at FDR < 0.05 using *cis*- and *trans*-eQTLs from eQTLGen (https://www.eqtlgen.org).

As part of the FUMA pipeline, we used these mapped genes to generate gene expression heatmaps using GTEx v8 (54 tissue types and 30 general tissue types). Gene expression values with a pseudocount of 1 were normalized across tissue types using winsorization at 50 and log2 transformed. Using the hypergeometric test, we tested for significant enrichment of our input genes in DEG sets for the different tissue types using a Bonferroni corrected p-value ≤ 0.05. Finally, we tested for overrepresentation of our input genes in predetermined gene-sets using hypergeometric tests. Gene-sets were obtained from MsigDB, WikiPathways, and GWAS-catalog reported gene-sets. We used Benjamini-Hochberg FDR correction for each of the categories within these gene-set sources separately using a threshold of 0.05 for our adjusted p value.

### Roadmap Epigenetic State Enrichment

Based on the Roadmap 15-core epigenetic state database (28), we used data obtained from 23 blood samples spanning 127 epigenomes to map the QTLs in the summary statistics to their respective epigenetic states. Epigenetic state information was available for bins of 200bp. We aggregated this information into four categories; active enhancer states (Enh, EnhG), active promotor states (TssA, TssAFlnk), all enhancer states (Enh, EnhG, EnhBiv), and all promotor states (TssA, TssAFlnk, TssBiv). We tested for enrichment using Fisher’s exact test based on the number of unique 200bp bins variants mapped to. This was done after filtering the QTL’s down based on their p-value using different thresholds (1×10^-5^, 1×10^-6^ and 1×10^-7^). Enrichment p values were obtained after FDR correction.

### Cytokine Production Enrichment

Using nominally significant QTL’s we linked COVID-19 risk alleles to pathogenically stimulated cytokine production risk alleles. We then clumped all variants within a 250kb, 500kb, or 1Mb window around the respective top COVID-19 risk allele at the 3p21.31 loci. We clumped using a stringent 0.01 LD threshold to make sure all associations were independent of each other. Lastly, a binomial test was performed to see whether the amount of down-regulated cytokine production associations was greater than the upregulated ones.

### Biomarker Measurements

Hospitalized patients with presumed COVID-19 disease were included in a prospective cohort between March and April 2020 at the Radboudumc (Nijmegen, the Netherlands). Disease severity was defined based on the patient’s need for intensive care at the time of sampling. The inclusion and clinical characteristics of this patient cohort have been previously described in detail (29). The basic characteristics of the studies samples are shown in **Table S15**. Plasma samples were collected from EDTA blood and stored at -80°C. The plasma concentrations of CCL25 were determined using the commercially available Inflammation panel from Olink Proteomics AB (Uppsala, Sweden). The procedure of this immunoassay was performed as previously described (30). CCL25 levels are expressed on a log2-scale as normalized protein expression (NPX) values and normalized using control samples to correct batch effects. Values under the detection threshold were replaced with the lower limit of detection. CCL25 levels were compared between severe COVID-19 patients (ICU ward = 18) and non-severe COVID-19 patients (non-ICU ward, N = 28) using a linear regression analysis with age and sex as covariates. CCL25 levels were measured every 2 days until an endpoint was reached (either patients left the hospital, or died of the disease).

According to the manufacturer’s protocol, plasma concentrations of CXCL16 were measured using commercially available enzyme-linked immunosorbent assays (ELISA, Invitrogen, Thermo Fisher Scientific) according to the manufacturer’s protocol, with a lower and upper detection limit of 0.055 and 40 ng/mL, respectively. Plasma concentrations of VWF were measured using a commercially available ELISA (Abcam, ab223864), with a lower and upper detection limit of 0.055 and 40 µg/mL, respectively. Values under or above the range of detection were replaced with the lower or upper detection limits, respectively. The basic characteristics of the studies samples are shown in **Table S16**. The Wilcoxon rank sum test was used to compare protein levels between 57 ICU patient and 102 non-ICU patients. The student’s *t* test was used to compare protein levels after excluding all samples are above upper detection limit.

### scRNA-Seq Analysis

Samples from COVID-19 patients were collected during the first wave of the pandemic from Berlin between March and July 2020 in Germany. The Berlin cohort consists of 25 mild and 29 severe COVID-19 patients and 22 controls samples from publicly available scRNA-seq data. The detailed clinical characteristics of those samples have been previously described (31). Gene expression levels were compared between severe patients and mild or healthy controls using FindMarkers functions in *Seurat* v3.2.2 (32) with Wilcoxon Rank Sum Test. Genes at least 10% expressed in tested groups and Bonferroni-corrected p-values < 0.05 were regarded as significant differentially expressed genes.

### Visualization

R package ggplot2 was used to generate bar charts, box plots and scatter plots. We applied an online tool Locus zoom to present genes within candidate loci. We used R package pheatmap to generate heat maps.

## Results

### COVID-19 Loci Are Enriched for Expression in Immune Organs, Chemokine Signaling Pathways, and Enhancer Regions

**Figure 2:**
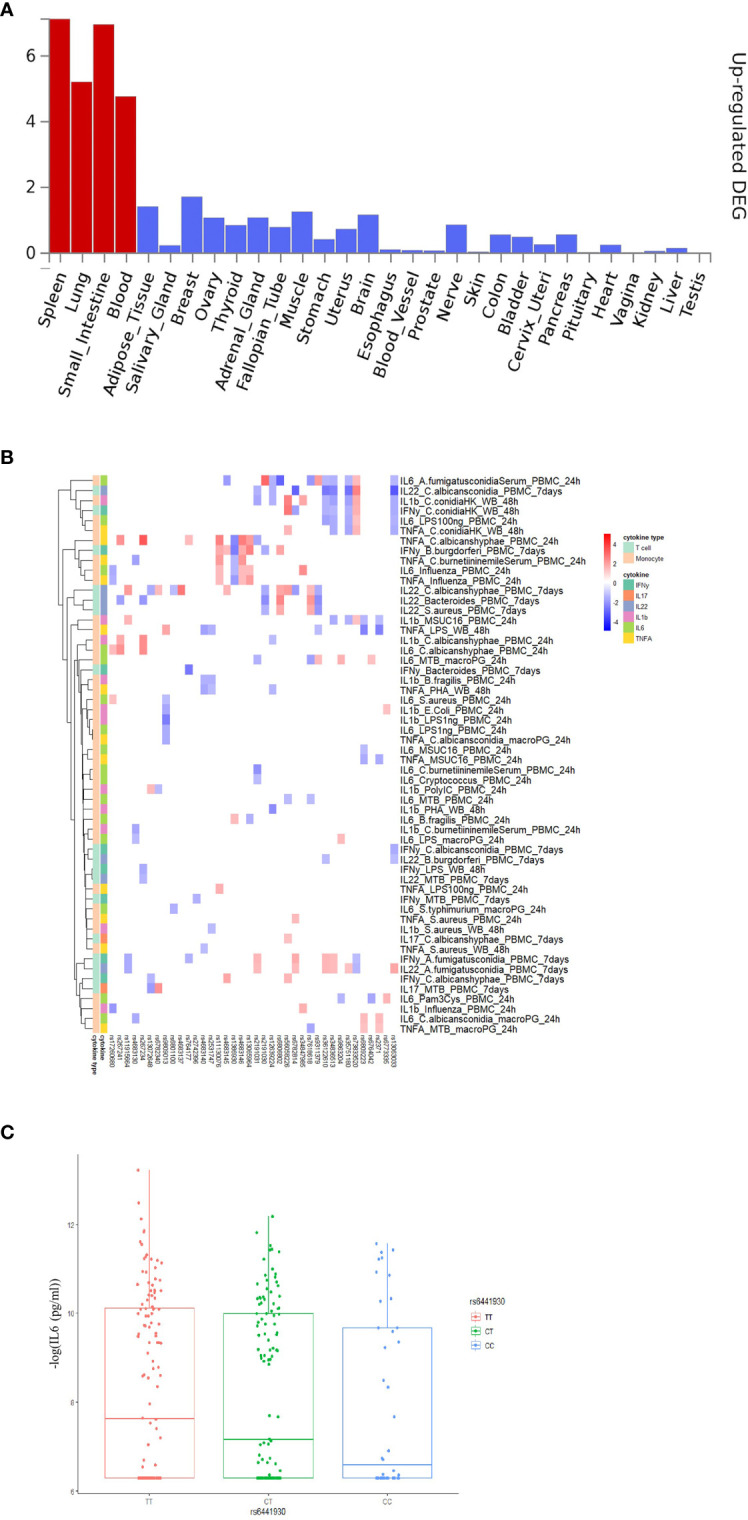
Functional annotation of COVID-19 loci from Severe COVID-19 GWAS study using the FUMA pipeline and association 3p21.31 loci with immune traits. **(A)** MAGMA Tissue expression results on 30 general tissues type (GTEx v8). FUMA analysis was done based on genes identified after using their genomic location, eQTL associations, and histone activity. Significant tissues are shown in red. **(B)** The heatmaps showing the 250kb window of independent association between 3p21.31 loci with cytokine production upon *in vitro* stimulations. Red color in heatmap indicates higher cytokine production led by risk allele in COVID-19 GWAS profiles, Blue color indicates lower cytokine production leaded by risk allele in COVID-19 GWAS profiles. **(C)** a boxplot showing COVID-19 risk allele(rs6441930-C) associated with reduced IL6 production with influenza stimulation of PBMC for 24 hours (p-value = 0.026).

To explore the functional impact of the identified COVID-19 loci, we firstly investigated if the genetic loci from the first COVID-19 GWAS study (performed by Severe COVID-19 GWAS Group) (15) are associated with any phenotypes available at the GWAS catalog (https://www.ebi.ac.uk/gwas/). Instead of limiting the analyses to the very few genome-wide significant variants, we used all suggestive associations (p-value < 10-5) as earlier proposed (33) in the first GWAS study, and subsequently correlated it with other immune traits. Among them, 211 loci were found to be associated with traits such as blood proteins/biomarkers, low-density lipoproteins (LDL) and very-low-density lipoprotein (VLDL) concentrations (**Table S1**). Subsequently, we performed functional annotation of significant loci and gene-mapping using the Functional Mapping and Annotation (FUMA) platform (27). The SNP2GENE function identified 32 independent SNPs located in 26 different loci (**Table S2**). Using multiple independent expression quantitative trait loci (eQTL) datasets, FUMA mapped 115 genes (**Table S3**) to these 26 genomic risk loci. Subsequently, using RNA-seq data of 30 tissues from the GTEx database (v8), we found that these genes mapped to suggestively significant COVID-19 QTLs and are significantly enriched for genes upregulated in immune organs such as the spleen and blood (**Figure 2A**). This, in turn, suggests that these tissues play an important role in the pathophysiology of COVID-19 (29, 34). Moreover, we also observed that mapped genes are mainly expressed in the small intestine and lung (**Figure 2A**), which are in line with the previous findings (35) that COVID-19 represents a multisystem illness and affects a wide range of organs, including respiratory and intestinal systems (36).

Pathway analysis of these 115 genes showed strong enrichment in chemokine binding and chemokine receptors binding (**Figure S1**), which is in line with the fact that chemokines can recruit immune cells to the site of infection and are critical for the function of the immune response (37). In addition, chemokine (C-C motif) ligand 20 (CCL20) has been reported as one of the most significantly elevated biomarkers in patients with severe COVID-19 in the intensive care unit (29). Furthermore, we also found a high number of enriched CD4 T cell-related pathways, specifically CD4 T cell counts, compared to other immune cells. T cells are thought to play an important role in providing long-term protection against SARS-CoV-2 reinfection, while severe COVID-19 is often associated with lymphopenia (38).

Considering that all significant SNPs that were identified by the COVID-19 GWAS in linkage disequilibrium (LD) with the 32 independent loci were significantly enriched in the non-coding intronic region (p-value = 0.036, Fisher’s exact test) (**Table S4**), we next examined whether the COVID-19 associated variants are enriched in regulatory DNA elements. We annotated all suggestively significant SNP’s (p-value < 10^-5^) based on their location with histone marks and chromatin states of 24 blood cell types in the Roadmap Epigenome Project (28).

We then tested the enrichment of any specific epigenetic regulatory state compared to all other states. We found that the COVID-19 genetic loci were strongly enriched for enhancer markers and weakly enriched in promoter markers (**Table S5**). These data suggest, although it remains to be demonstrated, that epigenetic mechanisms/regulation of immune responses may influence severity of COVID-19 infection, which is in accordance with other recently published studies (39). More work is warranted to investigate precisely the cellular targets and mechanisms involving these processes.

### 3p21.31 Locus Are Associated With Reduced Cytokine Production

Severe COVID-19 is characterized by complex immune dysregulation, combining defective immune features with hyperinflammatory innate immune traits (40, 41). However, these analyses in patients could be performed only late during the disease, and whether genetic risk for severe COVID-19 is characterized by low or high innate immune responses in a non-infected person is unknown. We, therefore, used the cytokine QTL (cQTL) data from the 500FG cohort (17) of the HFGP to test whether SNPs in 3p21.31 locus influence cytokine production upon stimulation. The cQTL data was obtained by associating genetic variants with cytokine response after *in vitro* stimulation of PBMC, macrophages, and whole blood cells with different pathogenic stimuli (17). We checked all SNPs located within a 50 kilobase window of top variant rs11385942 and observed that the risk alleles for a severe course consistently associated with lower cytokine production upon various *in-vitro* stimulations (**Figure S2** and **Table S6**). It is thus tempting to speculate that the people who carry risk alleles may not respond properly to initial virus infection, allowing for viral multiplication with subsequent high viral loads, leading to late systemic inflammation and poor outcome. However, many negative associations can be driven by linkage disequilibrium (LD). Therefore, we applied LD-based clumping and kept the most significant cQTL and checked all SNPs located within 250kb, 500kb and 1Mb window of rs11385942. Interestingly, we observed that the risk alleles for a severe course of COVID-19 are consistently associated with lower cytokine production upon various *in-vitro* stimulations at three *cis*-window sizes (p-value_250kb_= 0.02, p-value_500kb_ =2.43×10^-4^, p-value_1Mb_ = 2.57×10^-3^, **Figures 2B, C** and **Table S7**) and low monocyte-derived cytokine (tumor necrosis factor α (TNF-α), interleukin [IL]-1β (IL-1β), and IL-6) production upon various *in-vitro* stimulation at three cis-window size (p-value_250kb_ =0.02, p-value_500kb_ = 6.39× 10^-5^, p-value_1Mb_ =0.2, **Table S7**).

Later, we tested whether the COVID-19 risk SNPs are associated with the concentrations of circulating cytokines and levels of metabolites in the blood (**Tables S8, 9**). Using the same cohort, we found that IL-18 and IL-18BP concentrations show a suggestive positive association with the genetic risk of COVID-19 (**Figure S3**; **Table S10**). Additionally, one of the 3p21.31 loci, i.e., rs2191031, is suggestively negative associated with high-density lipoprotein cholesterol (HDL) (p-value =0.004, **Tables S8, S9**), which is consistent with previous findings that HDL concentrations were significantly lower in the severe COVID-19 disease group (42).

### Von Willebrand Factor (VWF) and Lymphocyte Function Are Strongly Influenced by the ABO Locus

**Figure 3:**
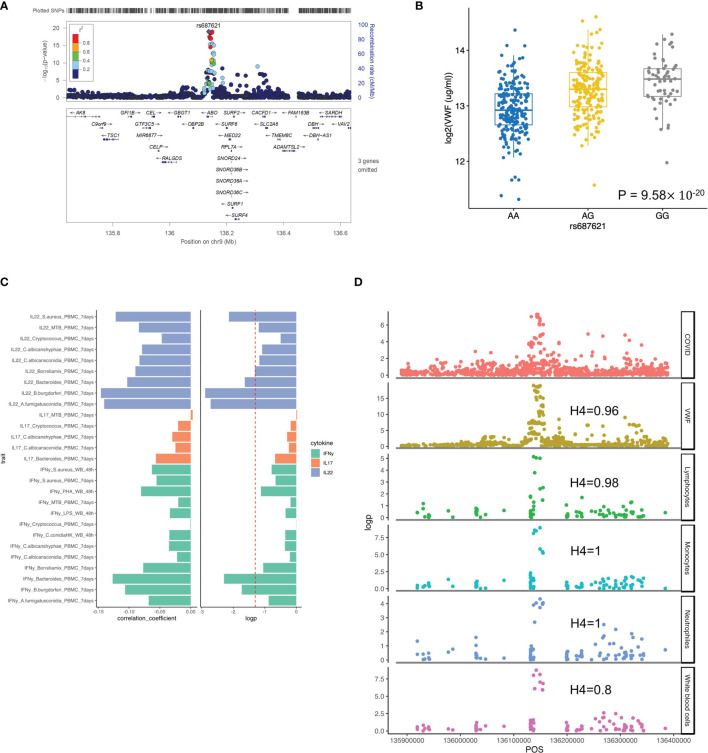
Functional annotation of ABO loci. **(A)** locus zoom plot showing the significant association between ABO loci and VWF level. **(B)** a boxplot showing COVID-19 risk allele(rs687621-G) associated with increasing VWF level (ug/ml) (p-value = 9.58×10^-20^). **(C)** a barplot showing consistent negative correlations between VWF levels and T cell-derived cytokines **(D)** scatter plots showing colocalization between ABO loci with VWF, lymphocytes, monocytes, neutrophils and white blood cell counts.

It is well established that the ABO blood group influences the plasma levels of VWF (43), and elevated VWF levels are associated with severe COVID-19 (44). We, therefore, tested the association of ABO locus with VWF circulating concentrations in the individuals of the 500FG cohort. Of note, we found the risk allele rs687621-G is significantly associated with elevated concentrations of VWF (p-value = 9.58×10^-20^) (**Figures 3A, B**). Recent studies have also reported that the VWF circulating concentration is highly related to COVID-19 severity (45, 46). As VWF concentration in plasma is an indicator of inflammation, endothelial activation and damage (47), our results suggest that the association of VWF and COVID-19 severity is very likely mediated through genetic regulation.

We next tested if this specific locus is associated with immune functions. Interestingly, we observed a consistently negative correlation of VWF risk allele and cytokine production derived from lymphoid cells (both T and NK cells) in response to various *ex-vivo* stimulations (**Figure 3C** and **Figure S4**). Among them, IL22 shows the strongest association, which may indicate that epithelial barrier functions modulated by IL-22 may be important in COVID-19 (48).” In addition, the ABO locus led by the variant rs687621 also showed statistically significant impact on several immune-mediated traits, including cell counts of lymphocytes (Coloc analysis H4: 0.98), monocytes (Coloc analysis H4: 1), neutrophils (Coloc analysis H4: 0.8), and whole blood cells (Coloc analysis H4: 1) (**Figure 3D**).

### No Association Between Genetic Risk Score of COVID-19 Severity With Sex and BMI

Polygenic risk scores (PRS) combine multiple risk alleles and capture an individual’s load of common genetic variants associated with a disease phenotype (49). Using the summary statistics provided in the GWAS study (15), we calculated the PRS for the samples from 500FG and 300BCG cohorts. Sex chromosome variants have been excluded in the PRS calculations to avoid potential bias (50). Since higher mortality of COVID-19 has been reported to be associated with male sex and BMI (13, 14), we investigated whether these host factors are associated with the PRS, a predictive measure of risk for development of severe COVID-19.

**Figure 4:**
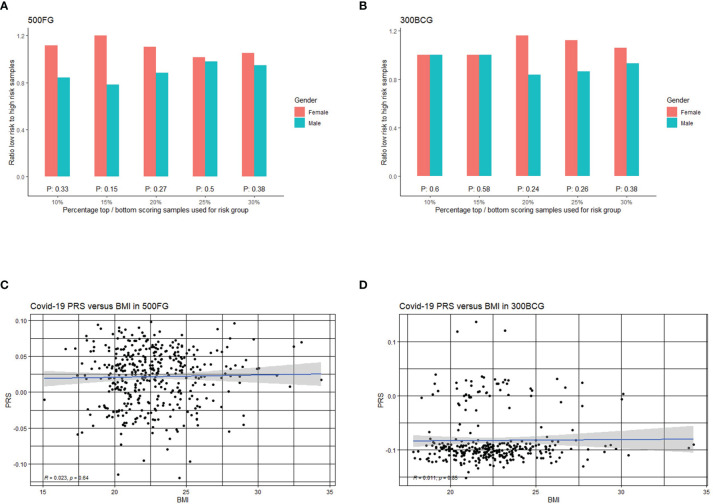
Correlation of COVID-19 PRS with sex and BMI. **(A)** Bar plot representing the ratio of low versus high PRS based risk between men and women in 500FG calculated without including the sex chromosomes. The X-axis shows the range of different quantiles (e.g.,10% corresponds to those individuals with PRS between 0^th^ and 10^th^ percentile of the population), and the Y-axis shows the odds ratio when comparing low PRS risk and high PRS risk in the male and female group from different quantiles. **(B)** Bar plot representing the ratio of low versus high PRS based risk between men and women in 300BCG calculated without including the sex chromosomes. **(C)** Scatter plot showing the correlation between PRS with BMI in 500FG. **(D)** Scatter plot showing the correlation between PRS with BMI in 300BCG.

At first, we assessed if men have a higher genetic risk compared to women in 500FG. Hereby, we defined people with top 10% PRS as a high-risk group and those with bottom 10% PRS as a low-risk group. As shown in **Figure 4A**, males tend to have a higher severe COVID-19 risk than females. We next used different percentile cut-offs (15%, 20%, 25% and 30%) to re-define low and high-risk groups. Interestingly, we observed a consistent pattern that men have equal or higher genetic risk (PRS) than women at different percentile cut-offs. These results can be replicated in a similar but independent cohort (300BCG cohort, **Figure 4B**). Meanwhile, the genetic risk difference between men and women can be attenuated when a loose cut-off has been defined. However, the meta-analysis of two cohorts showed no significant p-values at any of the percentile cut-offs (**Tables S11, S12**). Our data was not able to provide evidence that COVID-19 severity in men is genetically determined. However, such determination may be located on sex chromosome (51, 52) which are not modelled in our PRS calculation.

As obesity or overweight has been reported as a risk factor for serious illness or death from COVID-19, we also tested if the PRS is associated with BMI (**Figures 4C, D**). We did not observe any significant correlation between PRS and BMI.

### Association Between Severity Risk Factors and Cytokine Response Was Replicated in a Second GWAS Study

**Figure 5:**
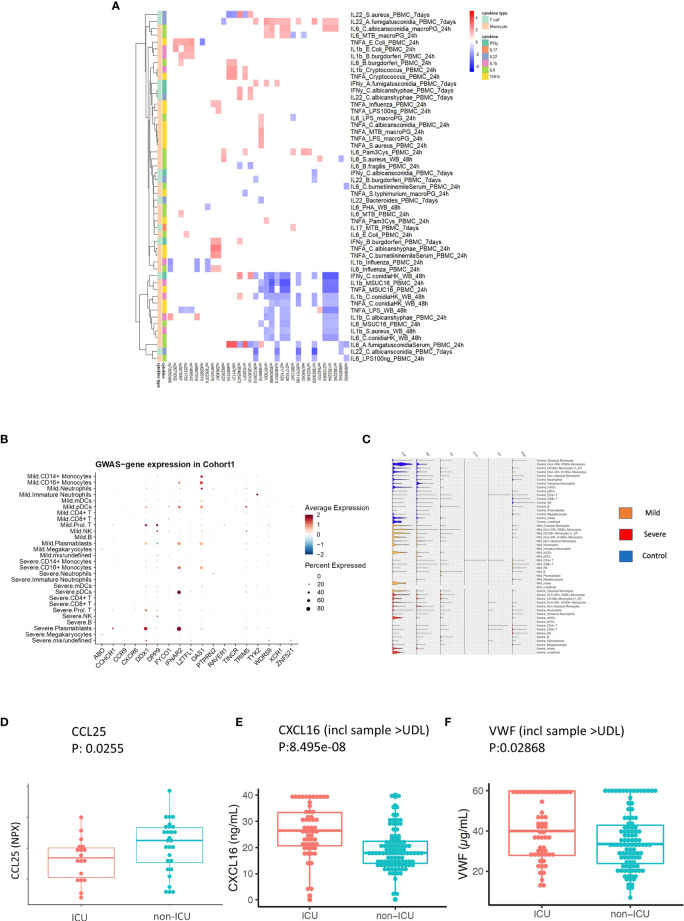
Replication and validation. **(A)** Heatmap showing the association between 3p21.31 locus and immune traits in GenoMICC study. The red color corresponds to higher cytokine production leaded by risk allele in COVID-19 GWAS profiles, whereas blue color indicates lower cytokine production leaded by risk allele in COVID-19 GWAS profiles. **(B)** Dot plots of expression of GWAS genes in single-cell transcriptomics of COVID-19 patients. The GWAS genes were selected from the Severe COVID-19 GWAS (1) and GenOMICC study (30). **(C)** Violin plots of the expression of monocyte-derived cytokine genes and T cell derived cytokine genes in COVID-19 Berlin cohort based on single cell RNA-seq data. **(D)** A boxplot of the differential protein levels [NPX (Normalized Protein eXpression)] of CCL25 between 18 ICU and 28 non-ICU COVID-19 patients from another independent cohort. **(E)** A boxplot of the differential protein levels of CXCL2 (ng/mL) between 57 ICU and 102 non-ICU COVID-19 patients. **(F)** A boxplot of the differential expression of VWF (ug/mL) between 57 ICU and 102 non-ICU COVID-19 patients.

To investigate whether our findings are only specific to one cohort, we implemented the same functional analyses on the recently published GenOMICC GWAS study (53). At first, we performed FUMA analyses with the same parameter settings as above, and the 135 mapped genes showed significant enrichment in the spleen, blood, lung and small intestine (**Figure S5**), which is consistent with our interpretation of the function of the genetic loci on immune organs. Next, we sought to test whether COVID-19 GWAS SNPs identified in GenOMICC studies influence cytokine production upon stimulation. Like above, here we also observed that risk alleles at 3p21.31 locus are consistently associated with lower cytokine production upon various *in-vitro* stimulations (**Figure 5A**; **Table S7** and **Table S13**). **Table S13** listed 816 nominally significant stimulation pairs SNP- cytokine (p-value <0.05). Notably, 777 out of 816 SNP- cytokine stimulation pairs are from 3p21.31 loci. As 3p21.31 locus is the major genetic risk factor explaining severity outcome (54), our result may indicate a potential mechanism of major genetic factor impacting the severity of COVID-19 through a defective immune response.

### Validation in Single-Cell Transcriptomes and Proteomics Data of COVID-19 Cohorts

To investigate in which cells those COVID-19 GWAS genes expressed, we utilized single-cell transcriptomics data from a COVID-19 German Berlin cohort (31) to illustrate the cell type-specific expression of COVID-19 risk genes (**Figure 5B**). Among 37 COVID-19 risk genes, 10 were significantly differentially expressed between severe and mild COVID-19. In general, the people with mild symptoms show higher expression of those risk genes than those with severe symptoms (p-value _χ2test_ =6.9×10^-13^) (**Figure S6**). Interestingly, *OAS1* (2’-5’-Oligoadenylate Synthetase 1) shows higher expression in patients with mild symptoms (**Figure 5B**), which agrees with the recent findings that higher plasma OAS1 concentrations are associated with reduced COVID-19 severity (55). We next tested the expression of myeloid cells-derived and lymphoid cells-derived cytokine genes in single-cell transcriptomics data. We found that myeloid cells-derived cytokine genes are stronger expressed in patients compared to lymphoid cells-derived cytokine genes, which may indicate that innate immune response is of significant importance in disease development (**Figure 5C**). Therefore, we further tested the longitudinal expression change of *IL-1β* and *TNF*-*α* using the data from the same cohort (**Figure S7**). Notedly, we found that patients with mild symptoms usually show a decreasing pattern of expression in cytokines (*IL-1β*, *TNF*-*α*) during the disease course (from high to low), while the severe symptom patients show an increasing pattern of expression in cytokine genes (*IL-1β*, *TNF*-*α*) with time (from low to high). Such a pattern may suggest that mild patients have a sufficiently good response in the first phase of the infection, which inhibits efficiently the viral replication, subsequently resulting in decreased inflammation. On the contrary, severe patients show defective activation of antiviral innate immunity in the first phase of the disease, leading to prolongation of the disease process with inefficient and deleterious inflammatory response in the end. This observation is in line with the recent findings of immunosuppression in severely affected patients (56).

CCR9 and CXCR6 have been identified as putative causal genes of the 3p21.31 locus (57, 58). Thus, we next validate whether two inflammatory proteins CCL25 and CXCL16, which are ligands for Chromosome 3 GWAS gene *CCR9* and *CXCR6*, respectively, are associated with disease severity. We measured CCL25 and CXCL16 protein concentrations in two different patient cohorts using Olink platform technology and ELISA, respectively (Methods). We observed a significantly lower expression of CCL25 (p-value = 0.0255) (**Figure 5D**), while a significantly higher expression of CXCL16 in ICU patients (p-value= 8.495e-08) (**Figure 5E** and **Figure S8**). These results are in line with the reported protective effect of *CXCR6* and the risk effect of *CCR9* in transcriptomic regulation (59). Interestingly, the longitudinal data from COVID-19 patients also showed that a clinical improvement is often associated with increased CCL25 concentration (**Figure S9**).

Additionally, we also measured VWF concentrations in 159 COVID-19 patients (Methods) and found they were significantly higher (p-value = 0.02868) in the ICU patients (**Figure 5F** and **Figure S10**). This further supported our findings that the ABO genetic risk is associated with VWF levels.

## Discussion

Understanding the pathophysiology of COVID-19 is urgently needed for designing novel preventive and therapeutic approaches against this severe disease. One important tool for identifying the most important mechanisms mediating the severity of a disease process is genomics: genetic variants that influence susceptibility or severity to disease are usually located in genetic loci that impact important mechanisms for that particular disease. Using the information of a recently published GWAS assessing the severity of COVID-19 (15), and the rich datasets available in the HFGP, we interrogated the mechanisms through which genetic variants associated with severe COVID-19 exert their effects.

Among the genetic loci associated with severe COVID-19, the 3p21.31 gene cluster has been reported to be robustly associated with COVID-19 severity (54) and it was inherited through introgression from Neanderthals (60). This locus is currently regarded as a marker of COVID-19 severity, but crucial information is missing: are the risk alleles in this locus (that encode several cytokines and chemokines) associated with a lower or higher cytokine production? The answer to this question is crucial for understanding COVID-19: a genetic risk associated with low cytokine production would imply that severe COVID-19 is the consequence of a relative immunodeficiency, while a high cytokine production associated with genetic risk would mean that severe COVID-19 is a genetic hyperinflammatory disease. In our study, the 3p21.31 genetic polymorphisms associated with a high risk of severe COVID-19 were consistently associated with lower production of cytokines. This important discovery has significant prophylactic and therapeutic consequences. On the one hand, it implies that improvement of immune responses in healthy individuals would decrease the probability that they undergo a severe form of COVID-19. On the other hand, this also implies that the dysregulated immune responses that have been described at later time points in patients with severe COVID-19 (31, 61) are likely the consequence of accelerated viral multiplication due to defective immune responses in the first phase of the infection, and subsequent inappropriate systemic inflammation due to high viral loads during the second (late) phase of the infection.

Several studies have shown that ABO blood types are associated with COVID-19 severity (62, 63) and susceptibility (63–65). It is still not well-known how the ABO locus regulates COVID-19 susceptibility. As ABO blood groups are also expressed on endothelial cells and platelets, it has been speculated that this effect may manifest itself *via* elevated plasma VWF concentrations (66). Our results provide evidence supporting this hypothesis, by showing that the risk alleles in the ABO locus are associated with high concentrations of VWF. Moreover, interesting associations have been found between polymorphisms in this locus and the number of various immune cell populations, especially lymphocytes, since lymphopenia is also consistently associated with severe COVID-19 (67). This suggests that genetic factors are relevant to the host thrombo-inflammatory response.

While our study sheds further light on how COVID-19 genetic risk affects the human immune system, there are several limitations of this study: firstly, due to different sets of stimuli used in measuring cytokine production to stimulations in the two healthy cohorts, we are not able to replicate our findings of genetic associations with cytokine responses from the 500FG cohort in the 300BCG cohort. Secondly, one limitation in our functional data is the exclusive European ancestry of the populations studied. The question, therefore, remains whether these findings would be similar in populations of different ancestries (e.g., African or Asian ancestry). Thirdly, in our analysis of the stimulated cytokine response, we make use of the 500FG cQTL data. This dataset was obtained in the years before the COVID-19 pandemic, which limits our capacity to study cytokine expression in response to SARS-CoV-2 itself, and more studies are warranted to evaluate this. Finally, our genetic score did not include the sex chromosomes: many immune genes are located on the X-chromosomes, and we cannot exclude a sex-biased genetic susceptibility for severe COVID-19 involving such genes, as recently shown for TLR7-deficiency in young men (51).

Collectively, our data demonstrate that genetic variability explains an important component of the increased susceptibility to severe COVID-19. The genetic risk for severe COVID-19 is associated with defective cytokine production capacity, dysregulated endothelial function, and defective lymphocytosis. By pointing to potential pathways to be engaged by future immunotherapies, these findings may contribute to the development of novel treatment and prevention strategies for severe COVID-19.

## Data Availability Statement

The datasets presented in this study can be found in online repositories. The names of the repository/repositories and accession number(s) can be found below: https://ega-archive.org/ under the accession numbers EGAS00001004571 and EGAS00001005348 and https://hfgp.bbmri.nl/, 500FG data.

## Ethics Statement

The studies involving human participants were reviewed and approved by Arnhem-Nijmegen Medical Ethical Committee (300BCG (NL58553.091.16) and 500FG (NL42561.091.12)). The patients/participants provided their written informed consent to participate in this study.

## Author Contributions

MN, C-JX, and YL designed the study. YK and XC performed statistical analyses supervised by YL and C-JX, MJ, SM, VK, VM, LB, QM, and LJ established the cohorts, helped with the data analysis and interpretation of results. BZ, VK, MKG, AN, IG, and NJ performed the validation. C-JX, MN, YL, YK, and XC wrote the manuscript with input from all authors. All authors contributed to the article and approved the submitted version.

## Funding

MN was supported by an ERC Advanced Grant (833247) and a Spinoza Grant of the Netherlands Organization for Scientific Research. YL was supported by an ERC starting Grant (948207) and a Radboud University Medical Centre Hypatia Grant (2018). C-JX was supported by Helmholtz Initiative and Networking Fund (1800167). This work was also partly supported by the German Federal Ministry of Education and Research, NaFoUniMedCovid19”—COVIM [01KX2021].

## Conflict of Interest

The authors declare that the research was conducted in the absence of any commercial or financial relationships that could be construed as a potential conflict of interest.

## Publisher’s Note

All claims expressed in this article are solely those of the authors and do not necessarily represent those of their affiliated organizations, or those of the publisher, the editors and the reviewers. Any product that may be evaluated in this article, or claim that may be made by its manufacturer, is not guaranteed or endorsed by the publisher.
