# Supplementary material for: The Genetic Risk for COVID-19 Severity Is Associated With Defective Immune Responses
Source: Front Immunol. 2022 May 12;13:859387. doi: 10.3389/fimmu.2022.859387 (PMC9133558; doi:10.3389/fimmu.2022.859387)
Supplement: Supplementary file 1 [file DataSheet_1.pdf]

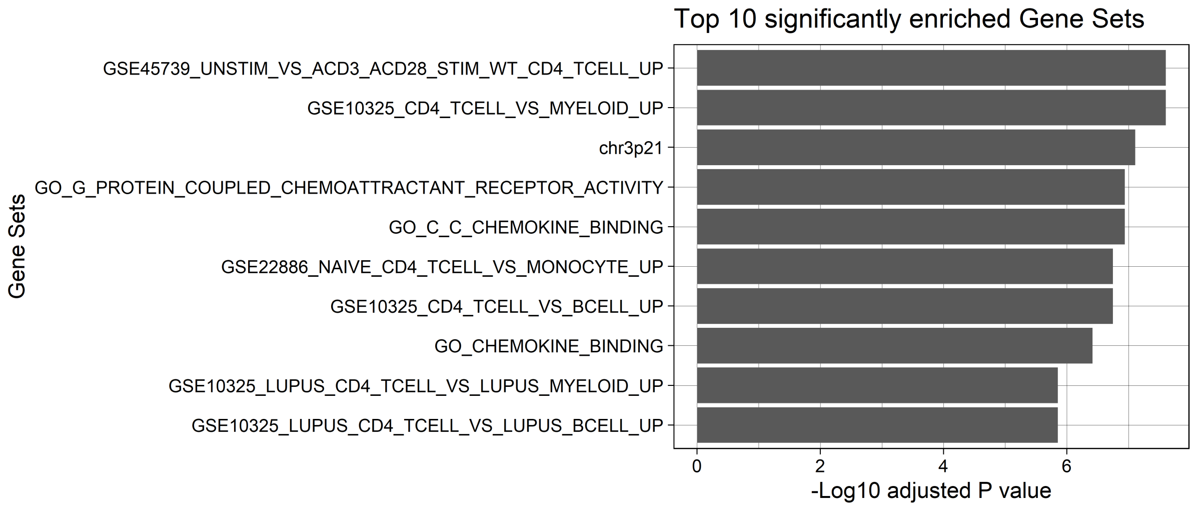

Fig. S1 A bar plot showing the top 10 significant enriched gene sets using functional annotation of COVID-19 loci by FUMA pipeline.

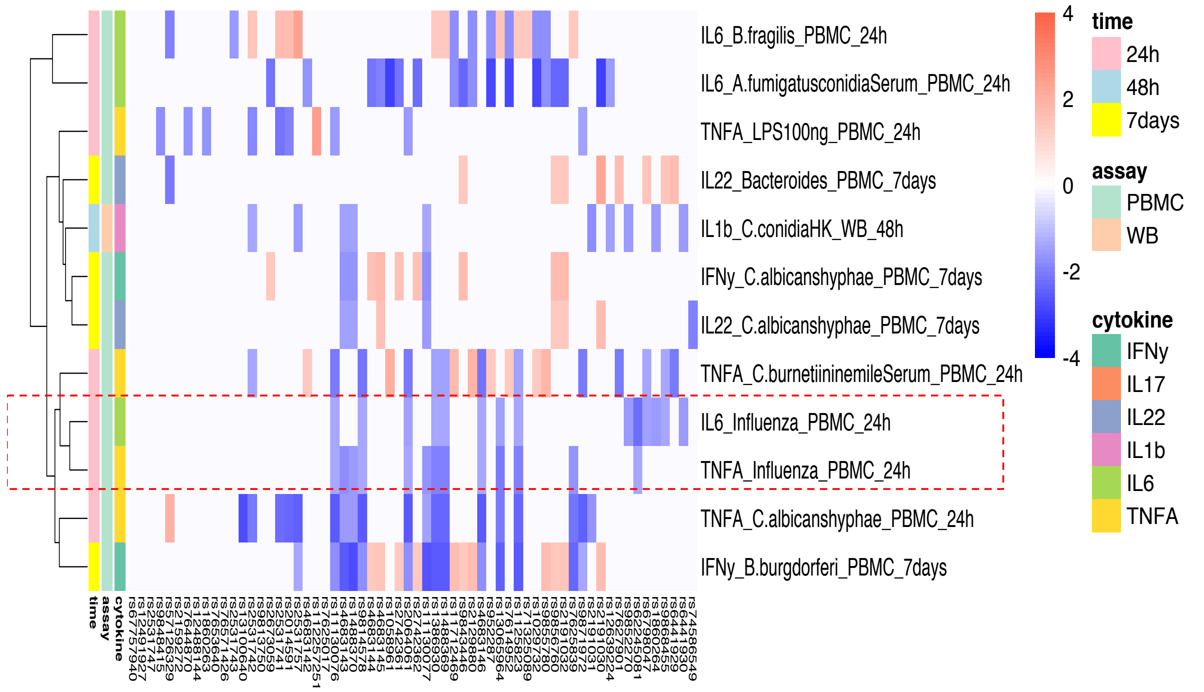

Fig. S2 The heatmap showing the association between 3p21.31 loci with cytokine production upon *in vitro* stimulations. Red colour in heatmap indicates higher cytokine production leaded by risk allele in COVID-19 GWAS profiles, Blue colour indicates lower cytokine production leaded by risk allele in COVID-19 GWAS profiles.

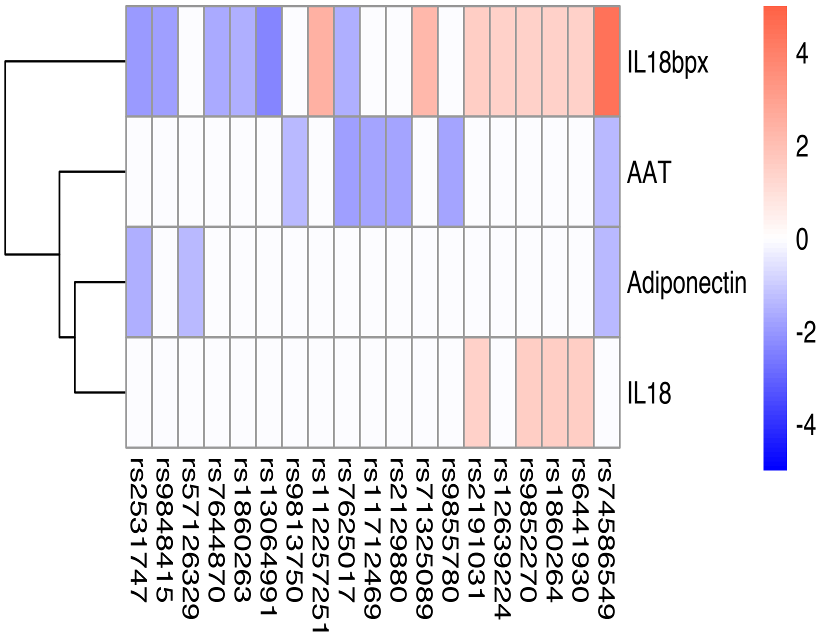

Fig. S3 Heatmap of the genetic associations between 3p21.31 loci and circulating mediator.

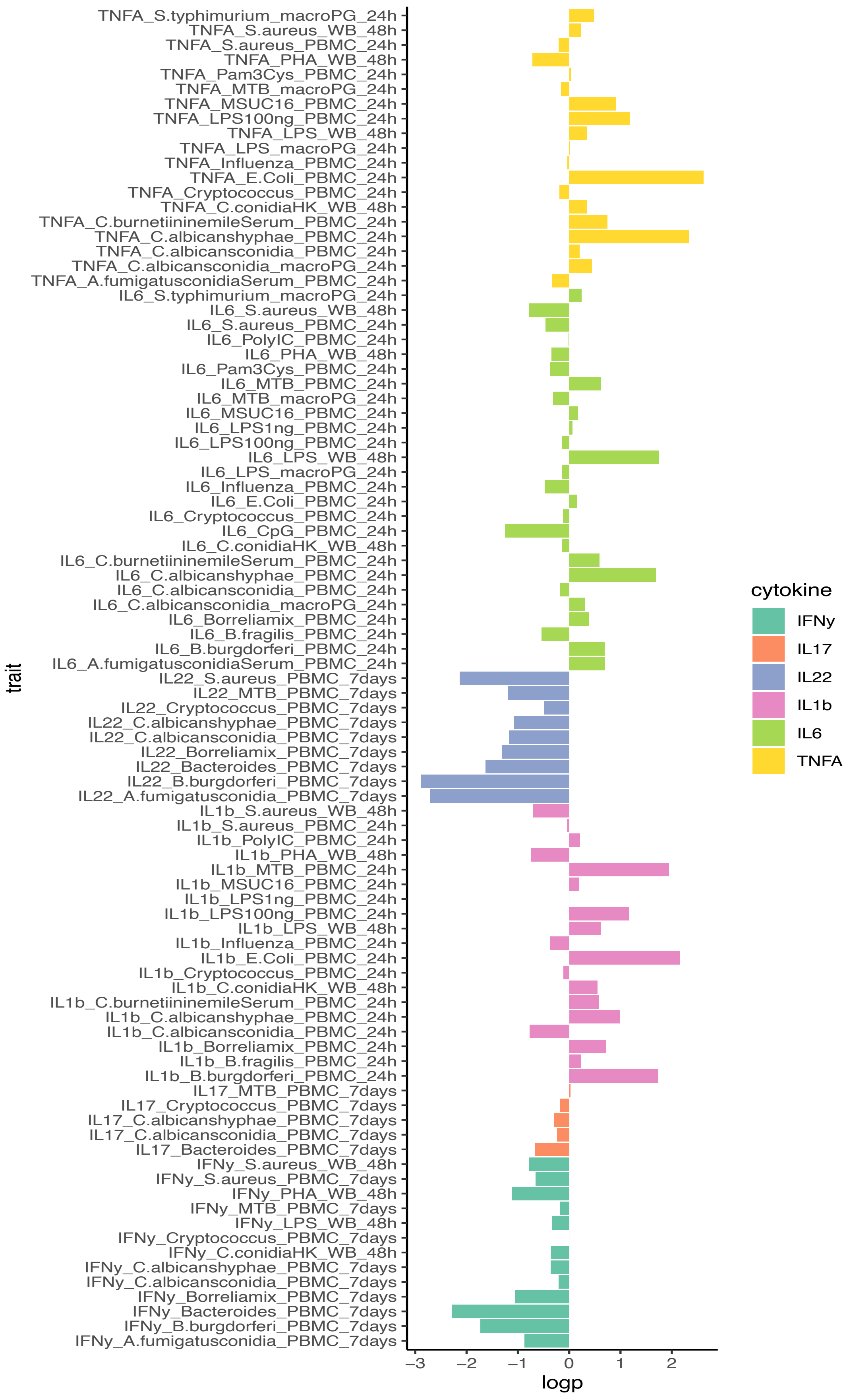

Fig. S4 A barplot showing associations between VWF levels and cytokine

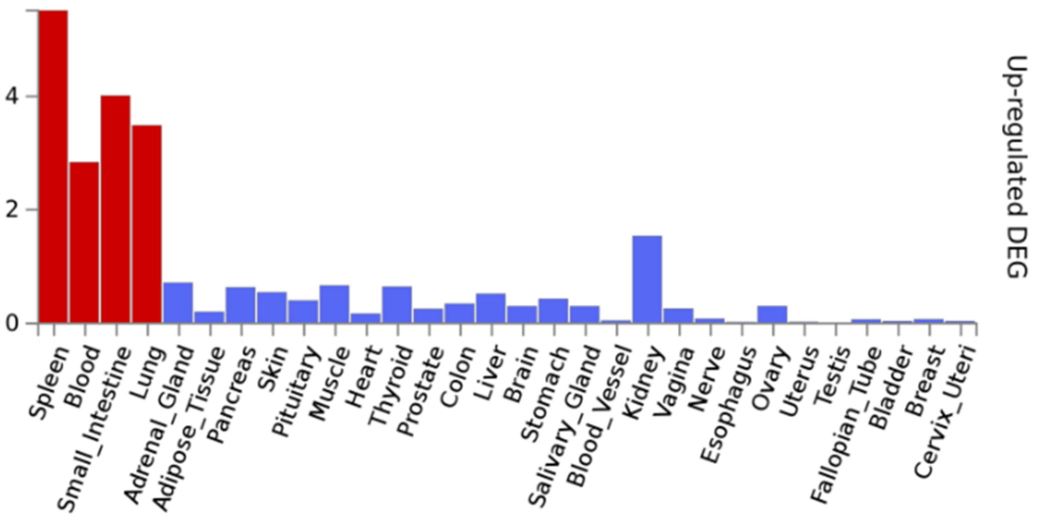

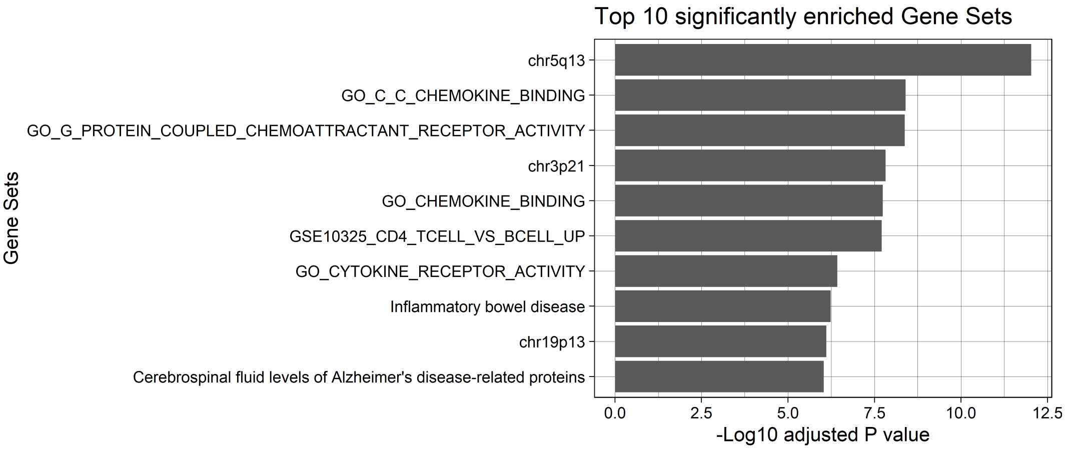

Fig.S5**.** Functional annotation of COVID-19 loci from GenOMICC study of European ancestry group using the FUMA pipeline. This was done based on genes identified after using FUMA to map QTLs based on their genomic location, eQTL associations, and histone activity. A) MAGMA Tissue expression results on 30 general tissues type (GTEx v8), B) The top 10 significant enriched gene sets.

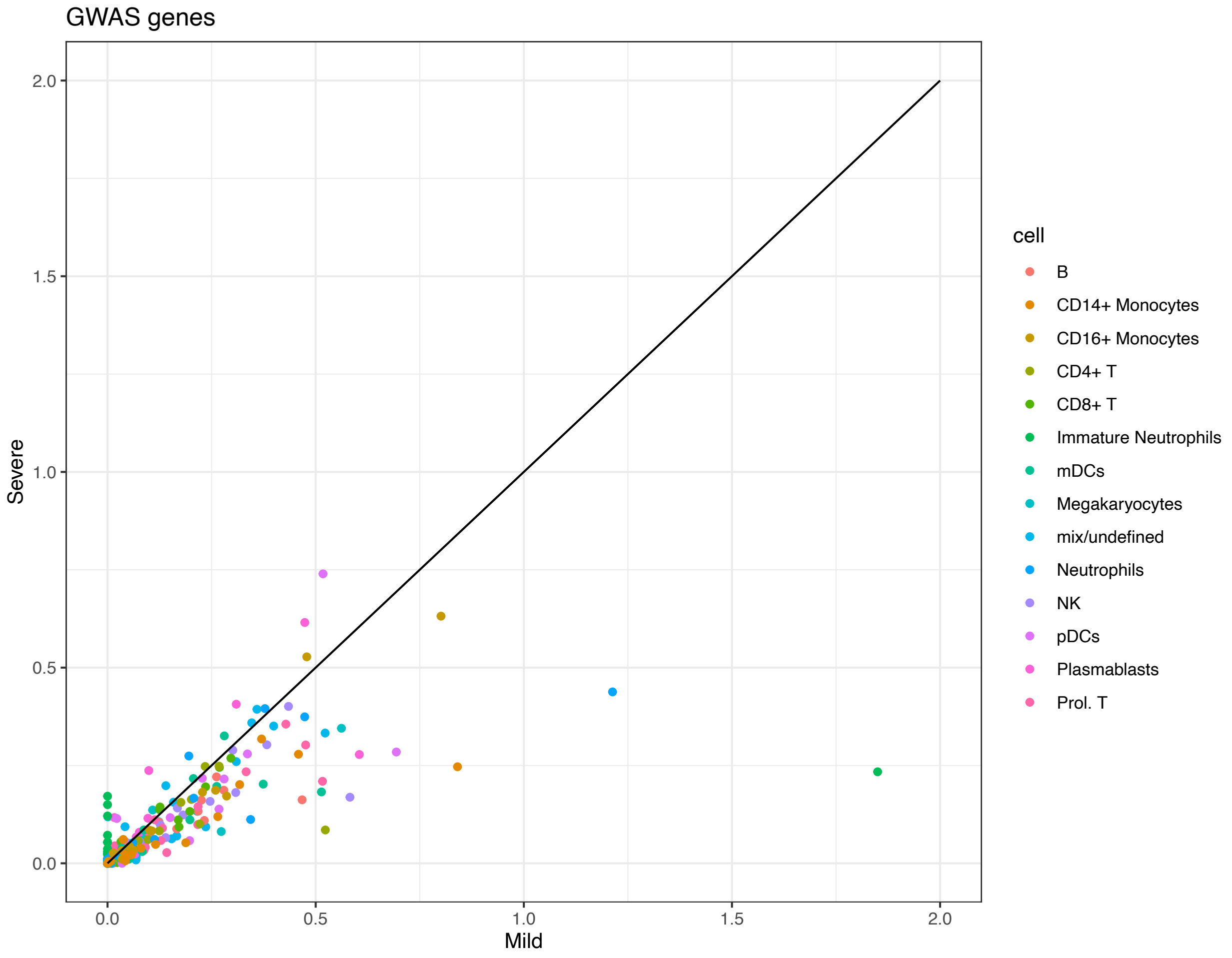

Fig.S6**.** A scatter plot showing the average cell type-specific expression of GWAS genes between mild and severe COVID-19 patients.

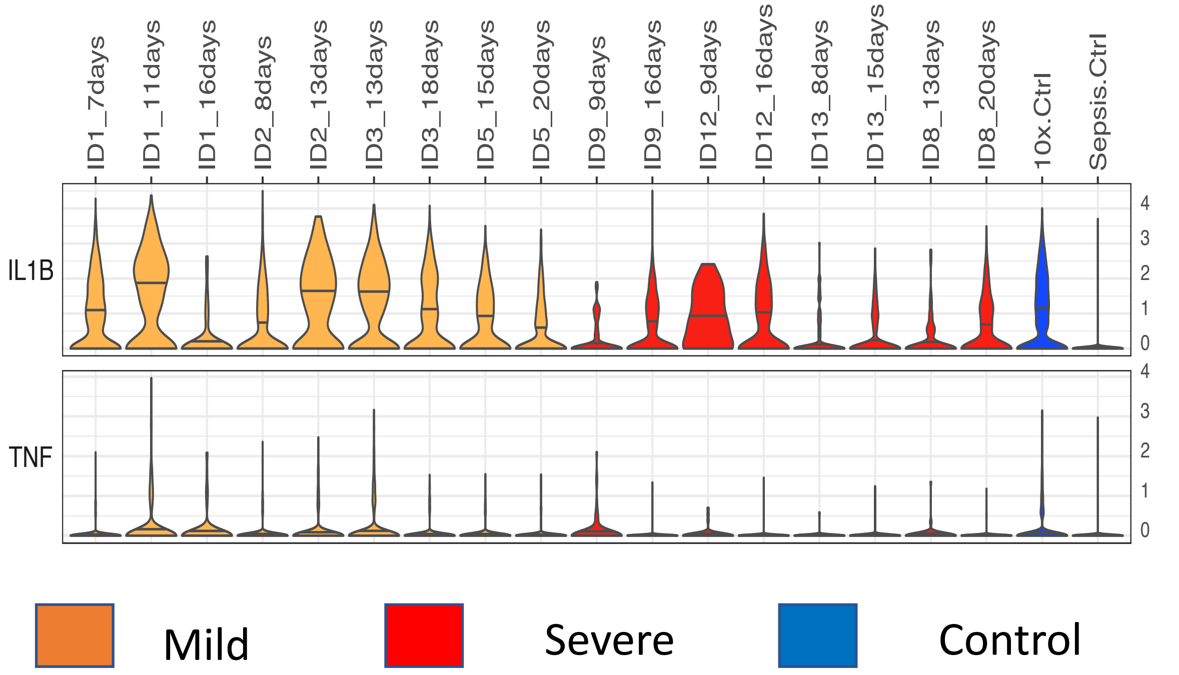

Fig. S7. The longitudinal change of *IL-1β* and *TNF-α* expression (violin-plots) in monocytes of COVID-19 Berlin cohort.

.

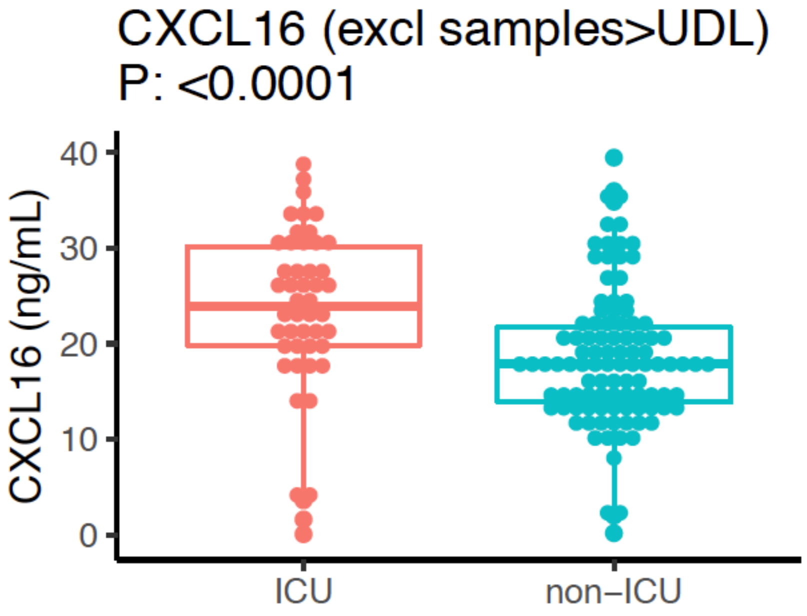

Fig. S8. A boxplot of the differential expression of CXCL16 between ICU and non-ICU COVID-19 patients after excluding samples which are above upper detection limit (UDL). The student's t test was used to test the difference here.

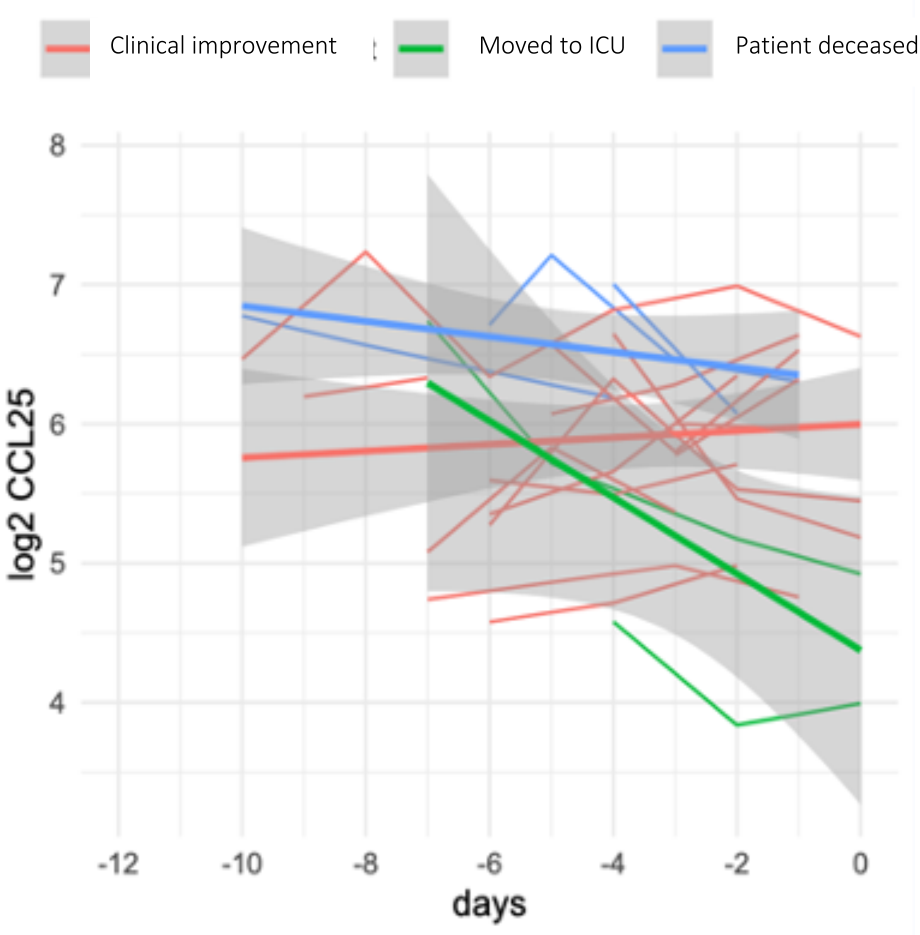

Fig. S9 The expression of CCL25 at different time points in three clinical groups (clinical improvement, moved to ICU and patient deceased).

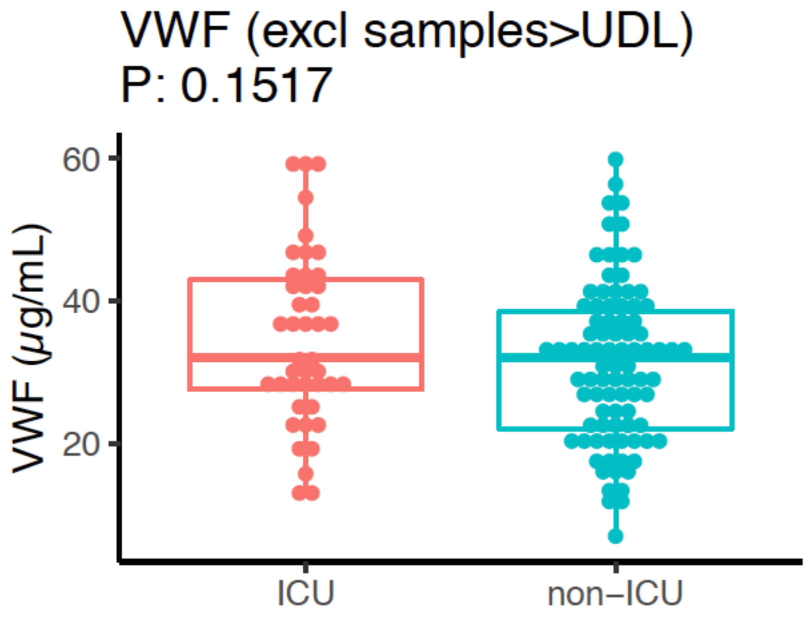

Fig. S10 A boxplot of the differential expression of VWF between ICU and non-ICU COVID-19 patients after excluding samples which are above upper detection limit (UDL). The student's t test was used to test the difference here.
